# IVIM Parameters on MRI Could Predict ISUP Risk Groups of Prostate Cancers on Radical Prostatectomy

**DOI:** 10.3389/fonc.2021.659014

**Published:** 2021-07-01

**Authors:** Chun-Bi Chang, Yu-Chun Lin, Yon-Cheong Wong, Shin-Nan Lin, Chien-Yuan Lin, Yu-Han Lin, Ting-Wen Sheng, Chen-Chih Huang, Lan-Yan Yang, Li-Jen Wang

**Affiliations:** ^1^ Department of Medical Imaging and Intervention, Chang Gung Memorial Hospital, College of Medicine, Chang Gung University, Taoyuan, Taiwan; ^2^ Department of Medical Imaging and Radiological Sciences, Chang Gung University, Taoyuan, Taiwan; ^3^ Department of Medical Imaging and Intervention, Chang Gung Memorial Hospital, Taoyuan, Taiwan; ^4^ Department of Clinical Science, General Electric (GE) Healthcare, Taipei, Taiwan; ^5^ Department of Medical Imaging and Intervention, New Taipei Municipal TuCheng Hospital, Chang Gung Memorial Hospital and Chang Gung University, Taoyuan, Taiwan; ^6^ Biostatistics Unit of Clinical Trial Center, Chang Gung Memorial Hospital, Taoyuan, Taiwan

**Keywords:** prostate cancer, IVIM, ISUP grade, diffusivity, pseudodiffusivity, perfusion fraction, kurtosis

## Abstract

**Purpose:**

To elucidate the usefulness of intravoxel incoherent motion (IVIM)/apparent diffusion coefficient (ADC) parameters in preoperative risk stratification using International Society of Urological Pathology (ISUP) grades.

**Materials and Methods:**

Forty-five prostate cancer (PCa) patients undergoing radical prostatectomy (RP) after prostate multiparametric magnetic resonance imaging (mpMRI) were included. The ISUP grades were categorized into low-risk (I-II) and high-risk (III-V) groups, and the concordance between the preoperative and postoperative grades was analyzed. The largest region of interest (ROI) of the dominant tumor on each IVIM/ADC image was delineated to obtain its histogram values (i.e., minimum, mean, and kurtosis) of diffusivity (D), pseudodiffusivity (D*), perfusion fraction (PF), and ADC. Multivariable logistic regression analysis of the IVIM/ADC parameters without and with preoperative ISUP grades were performed to identify predictors for the postoperative high-risk group.

**Results:**

Thirty-two (71.1%) of 45 patients had concordant preoperative and postoperative ISUP grades. D_mean_, D*_kurtosis_, PF_kurtosis_, ADC_min_, and ADC_mean_ were significantly associated with the postoperative ISUP risk group (all p < 0.05). D_mean_ and D*_kurtosis_ (model I, both p < 0.05) could predict the postoperative ISUP high-risk group with an area under the curve (AUC) of 0.842 and a 95% confidence interval (CI) of 0.726–0.958. The addition of D*_kurtosis_ to the preoperative ISUP grade (model II) may enhance prediction performance, with an AUC of 0.907 (95% CI 0.822–0.992).

**Conclusions:**

The postoperative ISUP risk group could be predicted by D_mean_ and D*_kurtosis_ from mpMRI, especially D*_kurtosis_. Obtaining the biexponential IVIM parameters is important for better risk stratification for PCa.

## Introduction

The Gleason scores (GSs) obtained from prostate biopsies or transurethral resection of the prostate (TURP) before radical prostatectomy (RP) are used as treatment guidance for prostate cancers (PCas) by stratifying them into low-risk (GS 6), intermediate-risk (GS 7) and high-risk (GS 8-10) groups. For example, low-risk patients may undergo active surveillance or brachytherapy as monotherapy (1, 2). However, the concordance of the GS on prostate biopsy and the GS according to RP are limited, ranging from 31% to 60% (3–5), which implies possibly inappropriate treatment selection for some patients when relying on the GS obtained from prostate biopsies (4, 5). Recently, the International Society of Urological Pathology (ISUP) adopted a new grading system for PCas using GSs 6, 3 + 4, 4 + 3, 8, and 9-10 as grades I, II, III, IV, and V, respectively, to replace the old risk stratification groups (i.e., GS 6, 7, 8-10) (6). The intermediate-risk group (GS 7) in the old risk stratification system is divided into GS 3 + 4 (grade II) and 4 + 3 (grade III) in the new ISUP grade system because there is a significant difference in recurrence between patients in the two new grades (6). The hazard ratios of biochemical recurrence relative to ISUP grade I were 1.9, 5.1, 8.0, and 11.7 for ISUP grades II, III, IV, and V, respectively. Thus, the accurate prediction of the postoperative ISUP grade according to the RP specimen is important for risk stratification and treatment selection for PCas.

Diffusion weighted imaging (DWI) is currently considered a key component of prostate multiparametric magnetic resonance imaging (mpMRI) examinations (7, 8). There are statistically significant correlations between the apparent diffusion coefficients (ADCs) and the GSs of PCas (9–11). The ADC values representing water diffusion are usually calculated from DWI using monoexponential fitting (12), which, however, does not consider the influence of intravoxel incoherent motion (IVIM) (13, 14). Thus, Le Bihan et al. (15). proposed an IVIM model using biexponential fitting, which allows the extraction of IVIM parameters, including diffusivity (D), pseudodiffusivity (D*), and perfusion fraction (PF). Two studies have shown that both ADC and IVIM parameters are associated with low risk (GS 6) and intermediate/high risk (GS 7-10) *via* biopsy or RP (16, 17). Nonetheless, it remains unclear whether the ADC/IVIM parameters are associated with the postoperative ISUP grades and thus may be useful for their prediction. In addition, for patients with preoperative ISUP grades obtained *via* biopsy or TURP, do the addition of the ADC/IVIM parameters have incremental value for risk stratification? Thus, the purpose of the current study was to elucidate whether ADC and IVIM parameters alone or in combination with preoperative ISUP grades could predict the postoperative ISUP grade.

## Materials and Methods

### Patients

**Figure 1:**
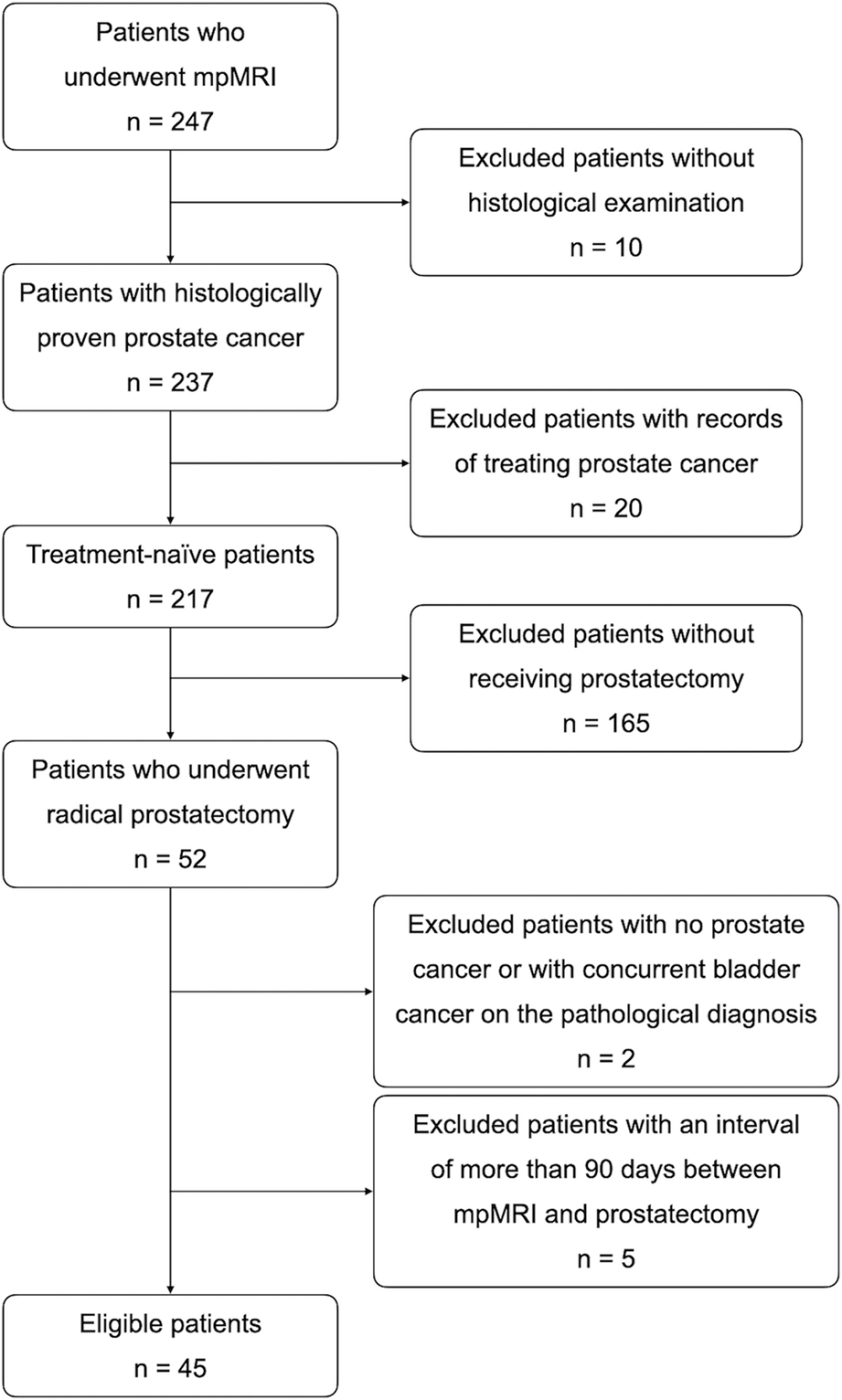
Flow diagram of the enrollment of the 45 patients selected by applying inclusion and exclusion criteria.

The institutional review board approved this retrospective study and provided a waiver for obtaining informed consent from the enrolled patients. From June 2016 to December 2017, 247 patients underwent prostate mpMRI, including DWI, IVIM, and dynamic contrast enhancement (DCE) pulse sequences. The patients who met all inclusion criteria and did not fit any of the exclusion criteria were enrolled for final analysis. The inclusion criteria were (1) a histological diagnosis of PCa by prostate biopsy or TURP, (2) no treatments for PCa before mpMRI, and (3) RP after mpMRI. At this stage, 195 patients were excluded due to violation of inclusion criteria, including ten without a histological diagnosis, 20 with records of treating PCa (such as prior RP, anti-hormone therapy, radiation therapy, etc.), and 165 without receiving RP. Of 52 patients who met all inclusion criteria, seven patients were excluded because of fitting the exclusion criteria. The exclusion criteria were (1) no PCa found in RP specimens (n = 1), (2) concurrent malignancy other than PCa in RP specimens (n = 1), (3) a time interval of more than 90 days between mpMRI and RP (n = 5) (16, 18), (4) poor diagnostic quality due to artifact of hip prostheses on mpMRI (n = 0), and (5) no detectable PCa on mpMRI (n = 0). Forty-five patients were eligible for this study and were used to construct the database (**Figure 1**).

### MRI Technique and IVIM/ADC Parameters on MRI

**Figure 2:**
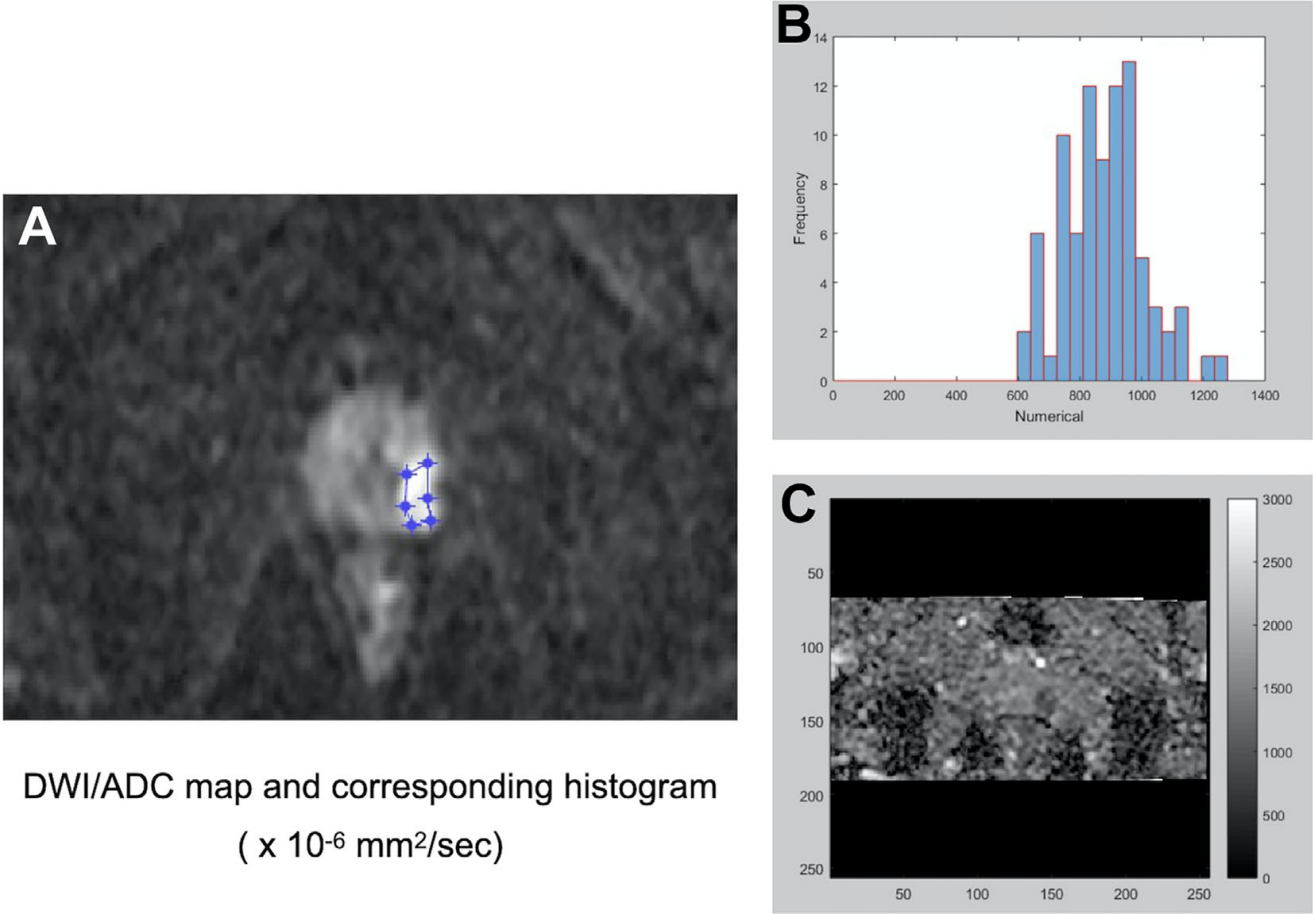
A representative PCa with a preoperative Gleason score of 3 + 3 for the histogram analysis of DW imaging measures. After identifying the dominant tumor nodule in the prostate gland, a region of interest (ROI) was delineated manually on a conventional DWI image (b = 1500 s/mm^2^) **(A)** to obtain an ADC map and the corresponding histogram **(B)** of the ADC map **(C)**.

**Figure 3:**
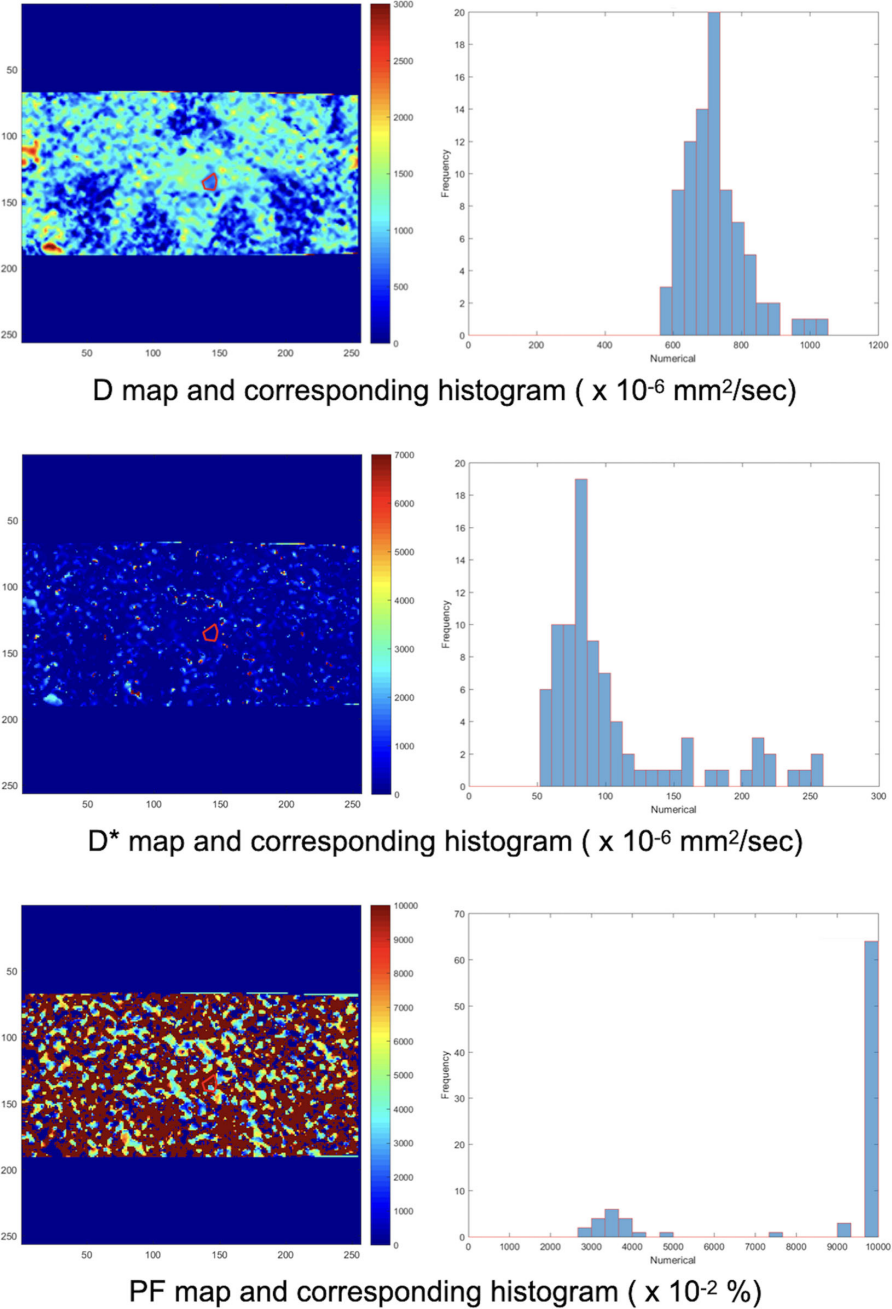
Another ROI was delineated manually on the IVIM D map. The ROI on the D map was automatically copied to the D* and PF maps by our homemade software. Then, the corresponding histograms of the D, D*, and PF maps were obtained. The process was repeated for each DWI image and IVIM map containing the dominant tumor nodule.

**Figure 4:**
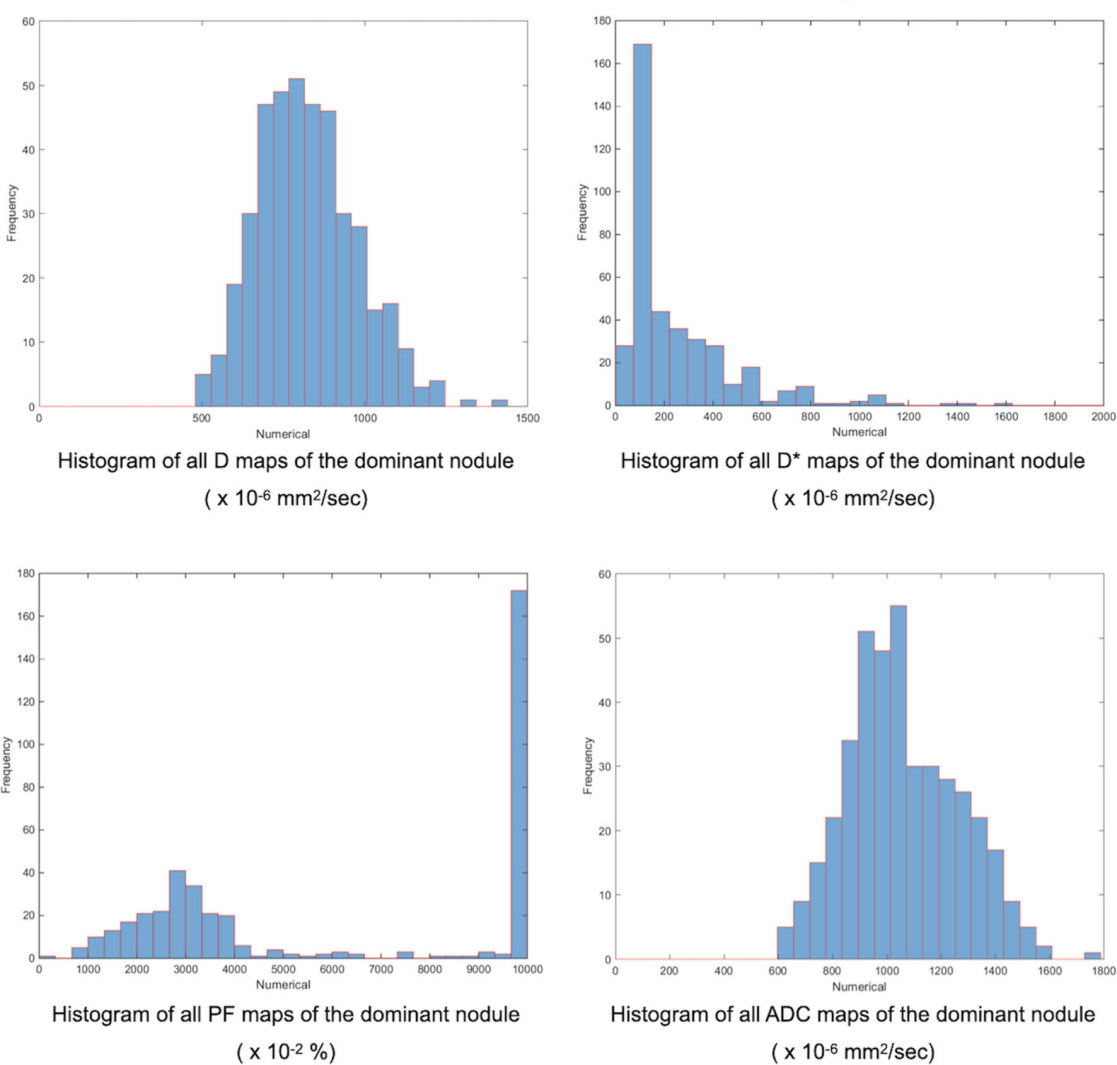
Finally, our homemade software constructed the whole dominant nodule histograms for D, D*, PF, and ADC by combining the different histograms from each image. The minimum, mean, and kurtosis of the IVIM/ADC parameters were extracted from the whole dominant nodule histogram and used for further analysis.

All MRI acquisitions were performed on a 3T clinical scanner (Discovery MR750, GE Healthcare, Milwaukee, USA). The mpMRI pulse sequences included conventional T2-weighted imaging (T2WI) in the sagittal, coronal and axial planes and T1-weighted imaging (T1WI) in the axial plane as well as functional imaging such as DWI, IVIM and DCE. T2WI was performed using a fast spin echo (FSE) sequence with repetition time (TR) = 5800-6100 ms; echo time (TE) = 92 - 103 ms; slice thickness = 4 mm; matrix = 384 × 320; and field of view (FOV) = 180 × 180 - 240 × 240 mm^2^. T1WI was performed using an FSE sequence with TR = 660 ms; TE = 15 ms; slice thickness = 4 mm; matrix = 256 × 224; and FOV = 180 × 180 mm^2^. IVIM imaging was performed using 8 b values (i.e., 0, 10, 30, 50, 80, 100, 400, 1000 s/mm^2^) with a reduced FOV (rFOV) = 20 × 10 cm^2^; matrix = 80 × 40; and TE = 53.4 ms. After IVIM DWI, DCE using a three-dimensional (3D) T1-weighted spoiled gradient-echo sequence in the axial plane (TR = 2.6 ms; TE = 1.1 ms; flip angle = 13°; number of excitations (NEX) = 1; matrix = 140 × 140; FOV = 280 × 280 mm^2^; and slice thickness = 4 mm) was acquired using a standard dose (0.1 mmol/kg body weight) of gadopentetate dimeglumine (Gd-DTPA; Magnevist; Bayer-Schering, Burgess Hill, UK) administered at a rate of 3 mL/s with a temporal resolution of 5.4 seconds and a total acquisition time of 324 seconds (60 phases). A uroradiologist with over 20 years of experience reviewed the ADC and IVIM images using homemade software written in MATLAB (R2015b; MathWorks, Inc., Natick, MA, USA) and delineated the largest region of interest (ROI) of the dominant tumor nodule on each image (**Figures 2**–**4**). Histogram values (i.e., minimum, mean, and kurtosis) of the IVIM parameters (D, D*, and PF) were then calculated and obtained using a biexponential model (15). Histogram values (i.e., minimum, mean, and kurtosis) of the ADCs generated from DWI using a standard monoexponential model (19) were recorded.

### Clinical Variables and Risk Groups Based on Preoperative and Postoperative Gleason Grading

For each patient, we recorded his age and prostate-specific antigen (PSA) titer at diagnosis. The preoperative ISUP grades of PCas (6, 20) were recorded using the histological results from transrectal ultrasound (TRUS) biopsy or TURP specimens and categorized into low-risk (grade I-II) and high-risk (grade III-V) groups. The percentage of positive TRUS biopsy specimen cores was recorded. The final pathological results based on RP specimens were then used for recording postoperative ISUP grades and similarly categorized into the two risk groups. The preoperative and postoperative ISUP grades and their corresponding risk groups of all patients were compared and recorded as same, upgraded or downgraded.

### Statistical Analysis

Descriptive statistics for continuous variables are expressed as the median and interquartile range (IQR) because of the small sample size and skewed distributions. Categorical variables are expressed as counts and proportions. The kappa statistic was calculated to analyze the agreement between the preoperative and postoperative ISUP grades. The associations of clinical characteristics and the IVIM/ADC parameters with the final risk groups (high/low) based on the postoperative ISUP grades were analyzed using the Mann-Whitney U test for continuous variables. Furthermore, multivariable logistic regression with forward selection procedure was performed to identify the predictors of a high-risk stratification for the RP specimens. First, all clinical characteristics (including age, PSA at diagnosis, positive biopsy specimen cores) and all IVIM/ADC parameters were initially entered in a logistic regression to identify the significant predictors of the postoperative ISUP risk group based on forward selection (Model I). Afterward, we added a factor of risk groups based on the preoperative ISUP grade combining with the model of forward procedure to investigate its adding effect (Model II). The ROC curves were plotted to show the predictive performance of the models. A similar multivariable logistic regression with forward selection procedure was also performed to identify the key predictors of postoperative ISUP grade or risk group upgrading. All statistical analyses were performed using SPSS Statistics version 25 (IBM, Armonk, New York). A two-tailed p-value of less than 0.05 was considered statistically significant.

## Results

The descriptive statistics of the clinical characteristics and the IVIM/ADC parameters of the 45 patients are summarized in **Table 1**. Before RP, the median PSA titer at diagnosis was 14.2 ng/mL, and 10 patients (22.2%) had PSA titer less than 9 ng/mL at diagnosis, ranging from 2.0 ng/mL to 8.3 ng/mL. Of these 10 patients, two patients (4.0%) had a PSA titer less than 4 ng/mL at diagnosis: 2.0 ng/mL and 3.2 ng/mL. Of the 45 patients, 41 (91.1%) underwent TRUS biopsy, and 4 (8.9%) underwent TURP to obtain the GS. Among ADC, D, and D*, D* had the lowest mean, and ADC had the highest. In contrast, D* had the highest kurtosis, and ADC had the lowest.

**Table 1:** Clinical characteristics and IVIM/ADC parameters obtained on MRI of 45 prostate cancer patients before radical prostatectomy.

Variables	Median (IQR)
Clinical characteristics	
Age at diagnosis (years)	66.0 (63.0–71.0)
PSA at diagnosis (ng/mL)	14.2 (9.1–20.4)
Positive biopsy specimen cores (%)	33.3 (8.3–50.0)
IVIM and ADC parameters	
D_min_ (×10^-6^ mm^2^/s)	481.0 (363.0–644.0)
D_mean_ (×10^-6^ mm^2^/s)	934.7 (832.7–1024.9)
D_kurtosis_	3.2 (2.6–3.8)
D*_min_ (×10^-6^ mm^2^/s)	0.0 (0.0–0.0)
D*_mean_ (×10^-6^ mm^2^/s)	376.6 (253.3–490.8)
D*_kurtosis_	44.4 (17.9–70.0)
PF_min_ (%)	0.02 (0.01–0.22)
PF_mean_ (%)	60.7 (54.6–72.1)
PF_kurtosis_	1.5 (1.3–2.4)
ADC_min_ (×10^-6^ mm^2^/s)	580.0 (445.0–852.0)
ADC_mean_ (×10^-6^ mm^2^/s)	1181.7 (1022.4–1281.1)
ADC_kurtosis_	3.0 (2.4–3.7)

All the statistics for the variables are expressed as the median (IQR).

IVIM, intravoxel incoherent motion; ADC, apparent diffusion coefficient; MRI, magnetic resonance imaging; PSA, prostate-specific antigen; D, diffusivity; min, minimum; D*, pseudodiffusivity; PF, perfusion fraction.

**Table 2** shows the distributions of the preoperative and postoperative ISUP grades of the 45 patients. For the preoperative TRUS biopsies and TURP specimens, ISUP grades I and III were the most common. However, grade III was the most common for the postoperative RP specimens. Fifteen of 45 (33.3%) patients had the same preoperative and postoperative ISUP grades. Thirty-two (71.1%) patients were categorized into the same preoperative and postoperative ISUP risk groups (16 low-risk and 16 high-risk). Overall, 7 (15.6%) patients upgraded from low risk to high risk (i.e., grade I to III for 2, and II to III for 5) and 6 (13.3%) patients downgraded from high risk to low risk (i.e., grade III to I for 1 and III to II for 5) postoperatively. There was moderate agreement between the preoperative and postoperative ISUP risk groups (kappa = 0.423, p=0.005). There were significant associations of preoperative and postoperative ISUP risk group, as 16 of 22 (72.7%) postoperative ISUP low-risk patients and 7 of 23 (30.4%) postoperative ISUP high-risk patients were regarded to have preoperative ISUP low-risk grades (p=0.005).

**Table 2:** ISUP grades of prostate cancers obtained before radical prostatectomy (RP) and using histological results of the RP specimens of the 45 patients.

Preoperative ISUP grades	ISUP grades from RP specimens				
	I	II	III	IV	V
I, n (%)	2 (15.4)	9 (69.2)	2 (15.4)	0 (0)	0 (0)
II, n (%)	1 (10.0)	4 (40.0)	5 (50.0)	0 (0)	0 (0)
III, n (%)	1 (7.7)	5 (38.5)	7 (53.8)	0 (0)	0 (0)
IV, n (%)	0 (0)	0 (0)	4 (66.7)	0 (0)	2 (33.3)
V, n (%)	0 (0)	0 (0)	1 (33.3)	0 (0)	2 (66.7)

Row percentages shown in parentheses.

ISUP, International Society of Urological Pathology.

**Figure 5:**
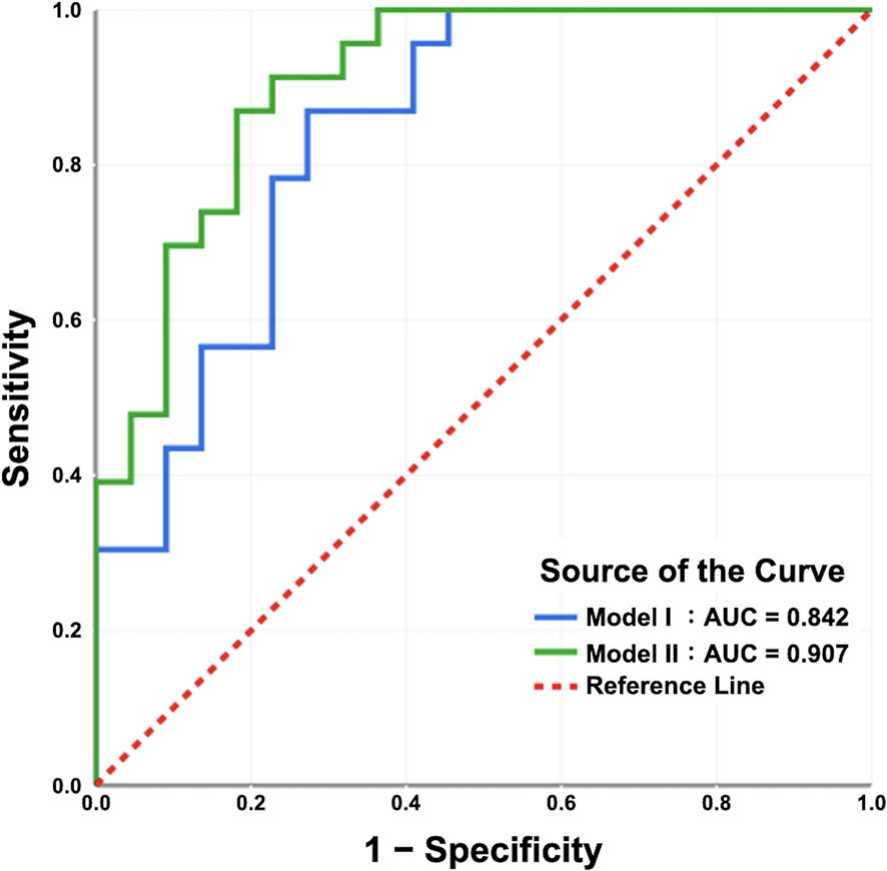
The receiver operating characteristic curves of the prediction models. The areas under the curves of model I and model II are 0.842 and 0.907, respectively. In model I, the IVIM/ADC parameters D_mean_ and D*_kurtosis_ were significant predictors for the ISUP high-risk group according to radical prostatectomy (RP) specimens. In model II, the use of the preoperative ISUP grade as an adjustment variable, in addition to D_mean_ and D*_kurtosis_, may enhance the predictive performance of the model.

The clinical characteristics (i.e., age and PSA at diagnosis and percentage of positive cores from TRUS biopsy) had no associations with both the postoperative ISUP high-risk group and postoperative ISUP risk group upgrading (**Table 3**; **Table S1** and **S2**). However, multiple IVIM and ADC parameters, including D_mean_, D*_kurtosis_, PF_kurtosis_, ADC_min_, and ADC_mean_, were significantly associated with postoperative ISUP risk group (all p < 0.05, **Table 3**). Besides, D_mean_, PF_kurtosis_, and ADC_mean_, were associated with postoperative ISUP risk group upgrading (all p ≤ 0.05, **Table S2**). Further multivariable logistic regression analysis showed that D_mean_ and D*_kurtosis_ were significant predictors for the postoperative ISUP high-risk group (both p < 0.05, model I, **Table 4**). D_mean_ was the only significant predictor for the postoperative ISUP risk group upgrading, with a negative relationship (p < 0.0001, **Table S3**). Significant predictors for the postoperative ISUP grade upgrading were not identified. By using the preoperative ISUP grade as an adjustment variable, the additive effect of D*_kurtosis_ and Dmean could improve the performance of the prediction models (p < 0.05, model II, **Table 4**). **Figure 5** shows that the areas under the ROC curves for model I and model II were 0.842 (95% CI 0.726–0.958) and 0.907 (95% CI 0.822–0.992), respectively.

**Table 3:** Associations of clinical characteristics and IVIM/ADC parameters obtained before radical prostatectomy with final risk groups of 45 prostate cancer patients.

Variables	ISUP grade groups*		*p*
	Low risk (N = 22)	High risk (N = 23)	
Age (years)	65.5 (63.0–71.0)	66.0 (61.0–71.0)	0.849
PSA at diagnosis (ng/mL)	11.8 (8.3–17.4)	14.5 (9.1–21.4)	0.586
Positive biopsy cores (%)	25.0 (8.3–50.0)	33.3 (12.5–50.0)	0.741
D_min_ (×10^-6^ mm^2^/s)	494.0 (350.0–692.0)	455.0 (363.0–563.0)	0.247
D_mean_ (×10^-6^ mm^2^/s)	971.7 (901.4–1113.6)	881.6 (800.3–995.7)	0.035
D_kurtosis_	2.8 (2.2–4.0)	3.5 (3.0–3.8)	0.073
D*_min_ (×10^-6^ mm^2^/s)	0.0 (0.0–0.0)	0.0 (0.0–0.0)	0.282
D*_mean_ (×10^-6^ mm^2^/s)	423.5 (251.4–603.5)	369.6 (298.0–420.8)	0.555
D*_kurtosis_	19.3 (4.8–49.4)	59.7 (34.2–84.8)	< 0.001
PF_min_ (%)	0.06 (0.01–2.2)	0.02 (0.01–0.13)	0.219
PF_mean_ (%)	58.1 (42.8–73.2)	62.0 (57.6–71.2)	0.376
PF_kurtosis_	2.3 (1.6–3.9)	1.3 (1.3–1.5)	0.001
ADC_min_ (×10^-6^ mm^2^/s)	705.5 (535.0–927.0)	535.0 (434.0–682.0)	0.044
ADC_mean_ (×10^-6^ mm^2^/s)	1274.7 (1084.8–1304.4)	1094.6 (1016.8–1231.4)	0.035
ADC_kurtosis_	2.7 (2.2–3.7)	3.4 (2.8–4.0)	0.077

All the statistics for the variables are expressed as the median (IQR).

All compared with the Mann-Whitney U test.

*Final ISUP grade groups using results of histological examinations of radical prostatectomies.

IVIM, intravoxel incoherent motion; ADC, apparent diffusion coefficient; ISUP, the International Society of Urological Pathology; PSA, prostate-specific antigen; D, diffusivity; min, minimum; D*, pseudodiffusivity; PF, perfusion fraction.

**Table 4:** Multivariable analysis of significant predictors of high-risk group according to radical prostatectomy specimens using logistic regression analysis.

Predictor	Estimate (S.E.)	OR (95% CI)	*p*
*Model I*			
D_mean_ (×10^-6^ mm^2^/s)	-0.002 (0.001)	0.998 (0.996–0.999)	0.003
D*_kurtosis_	0.045 (0.014)	1.046 (1.018–1.075)	0.001
*Model II*			
D_mean_ (×10^-6^ mm^2^/s)	-0.005 (0.001)	0.995 (0.993–0.998)	0.002
D*_kurtosis_	0.052 (0.018)	1.053 (1.016–1.092)	0.005
* Preoperative ISUP grades*			
I	*Reference*		
II	2.785 (1.249)	16.193 (1.399–187.381)	0.026
III, IV, V	2.575 (1.111)	13.126 (1.489–115.733)	0.020

S.E., standard error; OR, odds ratio; CI, confidence interval; D, diffusivity; D*, pseudodiffusivity; ISUP, the International Society of Urological Pathology.

## Discussion

This study shows that there is only moderate agreement in the preoperative and postoperative ISUP risk groups, with a final concordance of 71.1%. Two major factors may account for this limited concordance: (1) bias in the pathological evaluation and (2) sampling error from an underrepresented area (21). Previous studies have also reported similar but lower concordances for GS risk groups ranging from 31% to 60% (3–5). Although the higher concordance achieved in this study could be explained by the use of more biopsy cores (12, 13) than in previous studies (10 or fewer), 29% of patients who upgraded or downgraded postoperatively remained misclassified in the risk stratification and could have been potentially misled in the treatment selection if it had been based on the preoperative ISUP risk group alone.

There were significant associations of the postoperative ISUP risk groups with ADC_min_, ADC_mean,_ D_mean_, PF_kurtosis_ and D*_kurtosis_ (all p < 0.05) but not with the clinical characteristics in this study. Previous studies have also reported that ADC_mean_ and D_mean_ are associated with the GS risk group but not with clinical characteristics (16–18, 22–24). Since there is an inverse correlation between ADC_mean_ and GS 6-10 obtained from biopsies (19), the significant differences in ADC_mean_ using the monoexponential model and D_mean_ using the biexponential model between GS risk groups are reasonable and could be expected. However, the new ISUP grades differ from GS, as GS 7 is now categorized into two grades, ISUP grade II for GS 3 + 4 and ISUP grade III for GS 4 + 3, because of their substantial differences in recurrence. Shan et al. (18). showed that ADC_mean_, PF_mean_, and D_mean_ could differentiate GS 3 + 4 from GS > 3 + 4 according to RP with AUCs of 0.744, 0.726 and 0.732, respectively (all p < 0.05), which is similar to our results except for PF_mean_. In IVIM models, PF represents the proportion of water flowing in capillaries of the total water in a voxel, and D* represents water movement in the randomly oriented capillary network mimicking diffusion (15). This study showed that the postoperative ISUP high-risk group had significantly higher D*_kurtosis_ and lower PF_kurtosis_ than the low-risk group, which means that there are more outliers of the D* distribution and fewer outliers of the PF distribution (25). Thus, the postoperative ISUP high-risk grades tend to have markedly more heterogeneous water movement in the capillary network in voxels and a relatively more restricted range of PF than the low-risk group. The associations of these ADC and IVIM parameters with the postoperative ISUP risk group implies their potential usefulness in preoperative risk stratification and prediction for the postoperative ISUP grades, which have replaced the old GS system worldwide.

From a practical point of view, it is necessary to address whether the ADC/IVIM parameters could predict the final postoperative ISUP risk groups, and multivariate analysis with controlling variables showed that D_mean_ and D*_kurtosis_, rather than PF_kurtosis_ and ADC_mean_, were significant predictors for the postoperative ISUP risk group in this study. This means that lower D_mean_ and higher D*_kurtosis_ values predict the postoperative ISUP high-risk group with an expected high accuracy (0.842), as shown by the AUC, which is higher than the concordance (0.71) of the preoperative and postoperative ISUP risk group. The limited concordance between the preoperative and postoperative ISUP grades or GSs might result in the inappropriate selection of treatment. Thus, are the ADC and IVIM parameters helpful in filling this gap? This study shows that the addition of D*_kurtosis_ into the model using the preoperative ISUP grades has incremental value, achieving a high AUC of 0.907, which accounts for a 67.8% decrease in upgrading/downgrading the postoperative ISUP risk group with respect to the preoperative ISUP risk group. Thus, it is worth obtaining IVIM parameters using a biexponential model because unlike the ADC parameters, they are significant predictors of postoperative ISUP grade, both without and with biopsy/TURP information.

Le Bihan et al. (15). proposed the IVIM model by assuming a 2-compartment scenario and characterized the diffusion signals with a biexponential decay function. Since IVIM is an expanded form of DWI, it can be used for PCa detection in peripheral and transition zones of the prostate, just like monoexponential-fitted ADC. Previous studies had shown that the IVIM parameters were not superior to ADC in evaluating PCa in the transition zone (22) but might increase the diagnostic performance in detecting PCa in the peripheral zone (24). For tumor detection in the whole prostate, IVIM parameters and ADC might have comparable diagnostic performance (18). Overall, the biexponential-fitted IVIM did not add more information in tumor detection than traditional ADC. However, the IVIM parameters, as shown in the present study, would be beneficial to predict GS, aggressiveness, and postoperative ISUP risk group of PCa. The IVIM diffusion might, therefore, potentially influence the treatment selection of PCa.

There are limitations in the present work. First, this is a retrospective study of PCa patients undergoing RP with possible selection bias resulting from the recruitment of operable patients undergoing active surveillance, radiation therapy or hormone therapy by using the GS from biopsies/TURP as a reference for treatment selection. Another limitation is the small number of patients included in the present study due to the strict inclusion and exclusion criteria used, which, however, were implemented to ensure comparability between mpMRI and the RP specimens (e.g., patients with a delay of more than 90 days between mpMRI and RP were excluded). Future studies with prospective designs and large patient cohorts should be performed to confirm our results.

In conclusion, predicting the postoperative ISUP risk group with the use of histological information from biopsies/TURP could be unsatisfactory and sometimes misleading. It might be feasible and helpful to use the IVIM parameters Dmean and D*kurtosis from mpMRI alone to predict the postoperative ISUP risk group. The addition of D*_kurtosis_ to the preoperative ISUP grades has incremental value in the prediction of postoperative ISUP grades. Therefore, it is important to obtain IVIM parameters using a biexponential model for better risk stratification for PCa before surgery or other treatments.

## Data Availability Statement

The raw data supporting the conclusions of this article will be made available by the authors, without undue reservation.

## Ethics Statement

The studies involving human participants were reviewed and approved by the Institutional Review Board of Chang Gung Medical Foundation (IRB number, 202000712B0). The IRB approved a waiver for obtaining informed consent from patients/participants in this study.

## Author Contributions

Study concept and design: L-JW and L-YY. Acquisition, analysis, or interpretation of data: all authors. Drafting of the manuscript: C-BC, Y-HL, and Y-CL. Critical revision of manuscript: L-JW and L-YY. Statistical analysis: L-JW, L-YY, C-BC, and Y-CW. Administrative, technical, and material support: Y-HL, Y-CL, Y-CW, S-NL, C-YL, T-WS, and C-CH. Study supervision: L-JW and L-YY. All authors contributed to the article and approved the submitted version.

## Conflict of Interest

Author C-YL was employed by General Electric (GE) Healthcare.

The remaining authors declare that the research was conducted in the absence of any commercial or financial relationships that could be construed as a potential conflict of interest.

## Abbreviations

GS, Gleason score; TURP, transurethral resection of the prostate; RP, radical prostatectomy; PCa, prostate cancer; ISUP, International Society of Urological Pathology; DWI, Diffusion weighted imaging; mpMRI, multiparametric magnetic resonance imaging; ADC, apparent diffusion coefficient; IVIM, intravoxel incoherent motion; D, diffusivity; D*, pseudodiffusivity; PF, perfusion fraction; DCE, dynamic contrast enhancement; FOV, field of view; PSA, prostate-specific antigen; TRUS, transrectal ultrasound.
