# Supplementary material for: IVIM Parameters on MRI Could Predict ISUP Risk Groups of Prostate Cancers on Radical Prostatectomy
Source: Front Oncol. 2021 Jul 1;11:659014. doi: 10.3389/fonc.2021.659014 (PMC8282053; doi:10.3389/fonc.2021.659014)
Supplement: Supplementary file 1 [file Table_1.docx]

Supplement Table 1. Associations of clinical characteristics and IVIM/ADC parameters obtained before radical prostatectomy with ISUP grade upgrading of 45 prostate cancer patients

| **Variables** | **ISUP grades*** | | ***p*** |
| --- | --- | --- | --- |
|  | **No upgrade  (N = 27)** | **Upgrade  (N = 18)** |  |
| Age (years) | 67.0 (62.0－71.0) | 66.0 (63.0－73.0) | 0.44 |
| PSA at diagnosis (ng/mL) | 14.5 (9.3－20.4) | 10.9 (8.3－21.4) | 0.61 |
| Positive biopsy cores (%) | 33.3 (16.7－50.0) | 20.8 (8.3－54.2) | 0.49 |
| D_min_ (×10^-6^ mm^2^/s) | 474.0 (300.0－673.0) | 491.5 (369.0－580.0) | 0.60 |
| D_mean_ (×10^-6^ mm^2^/s) | 940.0 (832.7－1063.0) | 885.3 (820.5－1024.9) | 0.66 |
| D_kurtosis_ | 3.1 (2.6－4.4) | 3.2 (2.7－3.6) | 0.53 |
| D*_min_ (×10^-6^ mm^2^/s) | 0.0 (0.0－0.0) | 0.0 (0.0－0.0) | 0.61 |
| D*_mean_ (×10^-6^ mm^2^/s) | 414.5 (280.2－559.8) | 305.5 (222.7－402.4) | 0.07 |
| D*_kurtosis_ | 33.1 (17.0－70.0) | 45.9 (20.6－72.3) | 0.93 |
| PF_min_ (%) | 0.02 (0.01－0.16) | 0.1 (0.01－1.54) | 0.09 |
| PF_mean_ (%) | 57.9 (52.1－70.4) | 66.1 (60.7－72.8) | 0.06 |
| PF_kurtosis_ | 1.5 (1.3－2.3) | 1.6 (1.3－3.2) | 0.87 |
| ADC_min_ (×10^-6^ mm^2^/s) | 582.0 (492.0－853.0) | 534.0 (290.0－744.0) | 0.18 |
| ADC_mean_ (×10^-6^ mm^2^/s) | 1231.4 (1082.6－1304.4) | 1120.7 (1003.2－1217.9) | 0.07 |
| ADC_kurtosis_ | 2.9 (2.4－3.8) | 3.1 (2.6－3.7) | 1.00 |

All the statistics for the variables are expressed as the median (IQR).

All compared with the Mann-Whitney U test.

* Final ISUP grades using results of histological examinations of radical prostatectomies.

IVIM, intravoxel incoherent motion; ADC, apparent diffusion coefficient; ISUP, the International Society of Urological Pathology; PSA, prostate-specific antigen; D, diffusivity; min, minimum; D*, pseudodiffusivity; PF, perfusion fraction.

Supplement Table 2. Associations of clinical characteristics and IVIM/ADC parameters obtained before radical prostatectomy with ISUP risk group upgrading from low-risk to high-risk of 45 prostate cancer patients

| **Variables** | **ISUP risk groups*** | | ***p*** |
| --- | --- | --- | --- |
|  | **No upgrade  (N = 38)** | **Upgrade  (N = 7)** |  |
| Age (years) | 66.0 (63.0－71.0) | 66.0 (63.0－73.0) | 0.63 |
| PSA at diagnosis (ng/mL) | 12.1 (9.0－17.4) | 15.7 (10.3－26.9) | 0.21 |
| Positive biopsy cores (%) | 33.3 (15.8－50.0) | 8.3 (8.3－50.0) | 0.38 |
| D_min_ (×10^-6^ mm^2^/s) | 485.0 (350.0－673.0) | 455.0 (369.0－494.0) | 0.46 |
| D_mean_ (×10^-6^ mm^2^/s) | 958.1 (841.6－1063.0) | 845.8 (800.9－881.6) | 0.05 |
| D_kurtosis_ | 3.0 (2.6－4.0) | 3.6 (3.3－3.8) | 0.38 |
| D*_min_ (×10^-6^ mm^2^/s) | 0.0 (0.0－0.0) | 0.0 (0.0－0.0) | 0.96 |
| D*_mean_ (×10^-6^ mm^2^/s) | 383.2 (252.2－508.5) | 325.2 (283.0－402.4) | 0.64 |
| D*_kurtosis_ | 40.9 (17.0－72.3) | 44.4 (22.4－56.7) | 0.93 |
| PF_min_ (%) | 0.02 (0.01－0.64) | 0.07 (0.01－0.15) | 0.99 |
| PF_mean_ (%) | 58.9 (52.1－72.1) | 65.7 (60.7－71.2) | 0.33 |
| PF_kurtosis_ | 1.6 (1.3－3.2) | 1.3 (1.2－1.6) | 0.02 |
| ADC_min_ (×10^-6^ mm^2^/s) | 601.0 (467.0－859.0) | 523.0 (281.0－635.0) | 0.10 |
| ADC_mean_ (×10^-6^ mm^2^/s) | 1207.9 (1066.7－1287.4) | 1048.9 (1003.2－1146.7) | 0.03 |
| ADC_kurtosis_ | 2.9 (2.4－3.7) | 3.2 (2.9－4.2) | 0.26 |

All the statistics for the variables are expressed as the median (IQR).

All compared with the Mann-Whitney U test.

* Final ISUP risk groups using results of histological examinations of radical prostatectomies.

IVIM, intravoxel incoherent motion; ADC, apparent diffusion coefficient; ISUP, the International Society of Urological Pathology; PSA, prostate-specific antigen; D, diffusivity; min, minimum; D*, pseudodiffusivity; PF, perfusion fraction.

Supplement Table 3. Multivariable analysis of significant predictors of postoperative ISUP risk group upgrading from low-risk to high-risk

| **Predictor** | **Estimate (S.E.)** | **OR (95% CI)** | ***p*** |
| --- | --- | --- | --- |
| D_mean_ (×10^-6^ mm^2^/s) | -0.002 (0.001) | 0.998 (0.997－0.999) | <0.0001 |

Analysis was made using logistic regression with forward selection procedure.

ISUP, the International Society of Urological Pathology; S.E., standard error; OR, odds ratio; CI, confidence interval; D, diffusivity; D*, pseudodiffusivity.
